# Spatial regression and geostatistics discourse with empirical application to precipitation data in Nigeria

**DOI:** 10.1038/s41598-021-96124-x

**Published:** 2021-08-19

**Authors:** Oluyemi A. Okunlola, Mohannad Alobid, Olusanya E. Olubusoye, Kayode Ayinde, Adewale F. Lukman, István Szűcs

**Affiliations:** 1 Department of Mathematical and Computer Sciences, University of Medical Sciences, Ondo City, Ondo State Nigeria; 2 Faculty of Economics and Business, Institute of Applied Economic Sciences, University of Debrecen, 4032 Debrecen, Hungary; 3 Department of Statistics, University of Ibadan, Ibadan, Oyo State Nigeria; 4 Department of Statistics, Federal University of Technology, Akure, Ondo State Nigeria

**Keywords:** Climate sciences, Environmental social sciences, Mathematics and computing

## Abstract

In this study, we propose a robust approach to handling geo-referenced data and discuss its statistical analysis. The linear regression model has been found inappropriate in this type of study. This motivates us to redefine its error structure to incorporate the spatial components inherent in the data into the model. Therefore, four spatial models emanated from the re-definition of the error structure. We fitted the spatial and the non-spatial linear model to the precipitation data and compared their results. All the spatial models outperformed the non-spatial model. The Spatial Autoregressive with additional autoregressive error structure (SARAR) model is the most adequate among the spatial models. Furthermore, we identified the hot and cold spot locations of precipitation and their spatial distribution in the study area.

## Introduction

The ordinary least squared regression (OLS) has become a household name in many disciplines, especially when there is a need to investigate the cause and effect relationship between a response variable and one or more covariates^1^ . However, the reliability of OLS results depends on certain assumptions commonly called the “Gauss Assumption”. One of the stringent of these assumptions is that the error terms in the model should be independent. The violation of this assumption in the classical regression makes the inference on the coefficient to be invalid due to inflated standard error.

In real-life situations, this assumption of OLS is not attainable because observations located in space are related to their nearby units^2^. The quest for a new framework that accounts for dependence structure in the data to fill the vacuum in the classical regression led to spatial statistics. This study is motivated to discuss a simplified approach that accounts for spatial dependence in the regression model, illustrates spatial regression analysis, and applies the technique to investigate a linear relationship between precipitation and its likely predictors, namely northing, easting, and elevation^3,4^.

An eminent technique in spatial statistics is the model with spatially autoregressive factors either in the dependent variable or the error term. Through a Monte Carlo experiment^5^, used this model type to investigate the unbiasedness and consistency property of the model as against Ordinary Least Square (OLS). Asymptotically, the authors found that OLS and spatial model converged when the spatial effect parameter is negligible.

The necessity of a model that includes spatial effect is a new development in geography; however, it has been widely applied in many other fields in recent years. In climatology, statistics cannot be over-emphasized and mathematical statistics is a viable tool with wide application in climatology research^6^. They also reported that climatology, to a large degree, is studying the statistics of climate and have been described using several adjectives depending upon whether they define relationships in time (serial correlation, lagged correlation), space (spatial correlation, tele-connection), or between different climate variables (cross-correlation)^6^. It is a known fact that many fields of interest in climate experiments exhibit substantial spatial correlation. The spatial autocorrelation inherent in the data can be addressed by spatial statistics and other related approaches^7^.

Precipitation/rainfall is the climate variable that has been widely studied more than other climatic variables. It will continue to receive the interest of researchers as the ongoing process of global warming persistent, especially in the developing countries that are prone to climate change. The instability of climate is a threat to agricultural products, especially where there is dependence on agriculture for good livelihood. Most importantly, the subsistence with poor irrigation becomes unbearable^8^. It is needless to argue that every facet of human life is connected to precipitation and its variability, seasonality and extremity has a lot of consequences on humans and health of the plants^9,10^ . Excess precipitation can result in flooding, damage of structures, roadways, building, pollution of surface and groundwater^11,12^.

Researchers had made several attempts to determine the predictors of precipitation using several statistical methodologies in developed and developing countries. Accordingly, they established that precipitation increases with an increase in elevation, especially when used as a single predictor to enhance the precipitation patterns^13–18^. Precipitation is a complex phenomenon that is affected by many factors depending on geographical and topographical settings. In the geographical sense, reports show that the distribution of precipitation depends on slope, exposure, orientation and other derivatives of elevation^19,20^. Similarly, regional topographic variables such as distance to the Mediterranean, characterization of the general shape of the Alps, distance to corresponding features of the Alps were found to influenced heavy rains while local measures of topography (e.g. altitude, slope, or azimuth) were less influential^14^. A strong and positive relationship exists between the considered variables and precipitation because of the orographic effect of the mountain terrain^17^.

A study conducted in Kelantan state, Malaysia using multiple linear regression to determine dominant predictors of precipitation among easting, northing, elevation, slope and wind speed, showed that easting, northing and wind speed were the dominant predictors of precipitation^3^.

Studies on precipitation or rainfall modelling in Nigeria is rare. Most authors focused on predicting precipitation or rainfall using regression and artificial neural network^21^. Compared quadratic and Poisson regression with artificial models (multilayer feed-forward neural network, cascade feed-forward neural network, and radial basis neural networks to predict monthly rainfall in Jigawa State, Nigeria using average temperature, minimum temperature, maximum temperature, relative humidity, sunshine duration, solar radiation as predictors. They reported that both quadratic and Poisson regression performed better than the artificial models^22^. Compared the performance of linear regression and artificial neural network (ANN) to ensure reliable prediction of monthly rainfall, Kano, Nigeria. They used the dataset that covered thirty-seven (37) years (1981–2017) and was collected from Kano meteorological station. Southern Oscillation Index (SOI); Niño1 + 2, Niño3, Niño3.4 and Niño4 which are climatic indices commonly used in monitoring El Niño–Southern Oscillation (ENSO) were used as the predictors in both the linear regression and ANN. They considered climate indices used for monitoring namely; Southern Oscillation Index (SOI), as the predictors. This study showed that ANN had a predictive power that was higher than the linear model and they recommended that ANN should be used with ENSO indices in the prediction of monthly rainfall for the study area^23^. Used data obtained from the archives of the Nigerian Meteorological Agency (NIMET) for seasonal rainfall prediction in Bauchi State, Nigeria. They made used of monthly means of Sea Surface Temperature (°C), Air Temperature (°C), Specific Humidity, Relative Humidity (%) and Uwind (m/s) at surface different pressure levels, 750hpa, 800hpa, 1000hpa) from January to May for a period of 32 years (1986–2017) as predictors and they buttressed the findings of Ahmad and Mustapha (2018) that ANN had superior predictive power than multiple linear regression model. Similarly^24^, developed a model using ANN for the prediction of precipitation and evapotranspiration. The predictors considered in the model were a combination of some large-scale climate indices (El Nino Southern Oscillation (ENSO) and North Atlantic Oscillation (NAO)) and meteorological variables (average air temperature, maximum temperature, minimum temperature, mean speed, mean solar radiation, sunshine hours). They alluded to the fact that the meteorological variables and climatic indices were important in the prediction of standardized precipitation and evapotranspiration.

Despite the increasing attention on precipitation and inherent spatial correlation problems, limited study has employed spatial statistics analysis and regression modelling. Some studies, discussed precipitation mapping and spatial–temporal analysis using the time series approach with no attention to spatial regression representation^25–27^.

The current study takes off the existing works on precipitation and extends the scope using various exploratory data analyses and spatial regression models. Hence, this study proposes a robust approach to handling geo-referenced data and discuss its statistical analysis. It is hypothesized that the spatial models will provide a better fit than the OLS. Precipitation is used as a function of easting, northing, and elevation to verify the statement of the hypothesis. These predictors are selected based on the pieces of literature and the availability of data. It is expected that the study will offer salient information on the distribution of precipitation regarding the location in space and provide a guide for an informed decision on water management planning for agriculture and other purposes.

## Spatial data concept and model formulation

The conventional non-spatial sample of $n$ independent observations ${y}_{i}, i=1,\dots ,n$ that is linearly related to matrix $X$ is known to have a data generating process (DGP) of the form:

1 $$ \left. {\begin{array}{*{20}r} \hfill {y_{i} = X_{i} \beta + u_{i} } \\ \hfill { u_{i} \sim N\left( {0, \sigma^{2} } \right)} \\ \hfill {i = 1} \\ \end{array} } \right\} $$

This specification indicates that each observation has an underlying mean of ${X}_{i}\beta$ and a random component ${\mu }_{i}$. From the classical point of view, for a situation where $i$ represent regions or points in space the observed values at one location (or region) are independent. Alternatively, statistically, independent observations imply that $E\left({u}_{i}{u}_{j}\right)=E\left({u}_{i}\right)E\left({u}_{j}\right)=0$. The assumption of independence greatly simplifies models but in spatial contexts, this simplification seems unattainable.

Conversely, “*spatial dependence* reflects a situation where values observed at one location or region depend on the values of *neighbouring* observations at nearby locations”. In this case, if observations $i=1$ and $j=2$ represent neighbours (perhaps regions with borders that touch), then there will be a situation which suggests a simultaneous data generating process of the form:

2 $$ \left. {\begin{array}{*{20}l} {y_{i} = \alpha_{i} y_{j} + X_{i} \beta + u_{i} } \hfill \\ {y_{j} = \alpha_{j} y_{i} + X_{j} \beta + u_{j} } \hfill \\ { u_{i} \sim N\left( {0, \sigma^{2} } \right), i = 1} \hfill \\ { u_{j} \sim N\left( {0, \sigma^{2} } \right), j = 2} \hfill \\ \end{array} } \right\} $$

This assertion emanates from the fact that the value assumed by ${y}_{i}$ depends on that of ${y}_{j}$ and vice versa.

The very notion of spatial dependence indicates the need to ascertain which other units in the spatial system have an impact on the particular unit under concern. Properly, this is conveyed in the topological notions of a neighbourhood. This quantification of the locational aspect of our sample data can be done in several ways. The contiguity based neighbourhood such as rook (common side), bishop (common vertex) or queen (common side or vertex) is a common form of representation.

**Figure 1:**
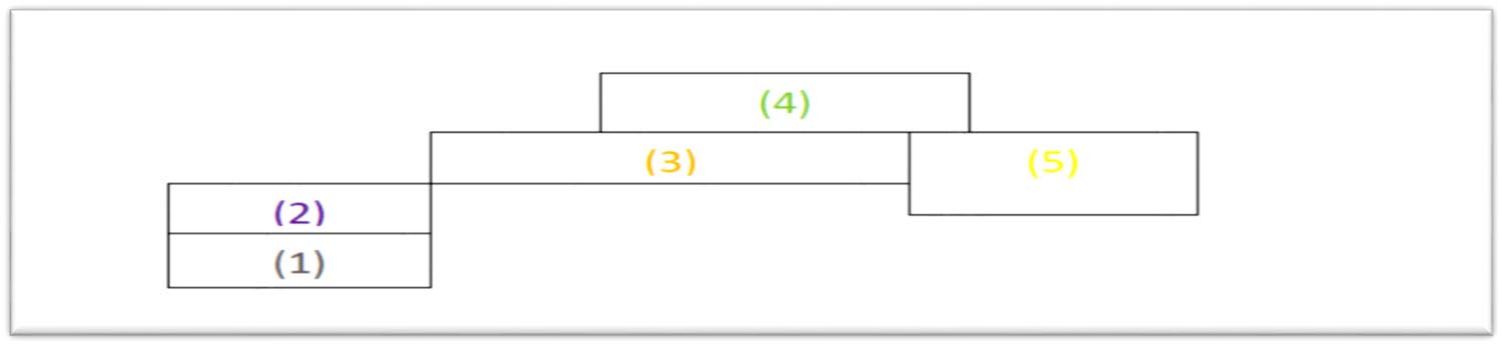
An illustration of the contiguity-based neighbourhood.

Figure 1 illustrates the definition of the various contiguity-based neighbourhood between sites ${s}_{i} and {s}_{j}$ while Table 1 gives a queen contiguity relation among the five regions.

**Table 1:** A five regions queen contiguity relation.

Region	Neighbours
1	2
2	1,3
3	2,4,5
4	3,5
5	3,4

From Table 1, region 2 is a neighbour to region 1 and by the symmetric property, region 1 must be a neighbour to region 2. Similarly, regions: 1 and 3; 2, 4 and 5; 3 and 5; and 3 and 4 are neighbours to region 2, 3, 4 and 5, respectively. These give rise to the spatial weight matrix, W which reflects the first-order contiguity relation among the five regions. The W is expressed as:

$$ {\text{W}} = \left[ {\begin{array}{*{20}c} 0 & 1 & 0 & 0 & 0 \\ 1 & 0 & 1 & 0 & 0 \\ 0 & 1 & 0 & 1 & 1 \\ 0 & 0 & 1 & 0 & 1 \\ 0 & 0 & 1 & 1 & 0 \\ \end{array} } \right] $$

The W is symmetric, and it has zeros on the main diagonal. This is done to prevent a unit from being a neighbour to itself. The spatial weights matrix is row-standardized to have row-sums of unity and produce a spatially weighted average term Wy of the dependent variable in the spatial lag model. Consequently, the spatial parameter associated with Wy has an instinctive interpretation of spatial autocorrelation coefficient; and also accelerates the maximum likelihood (ML) estimation of spatial models. Consequently, row-standardization has become a meeting in practice without further investigation. However, it may not be appropriate in some situations. Hence, the standardized W is given as:

3 $${W}^{s }={w}_{ij }/ \sum_{j}{w}_{ij} such that \sum_{j}{w}_{ij}^{s}=1$$

$$ {\text{W}}^{{\text{s}}} = \left[ {\begin{array}{*{20}c} 0 & 1 & 0 & 0 & 0 \\ {{1 \mathord{\left/ {\vphantom {1 2}} \right. \kern-\nulldelimiterspace} 2}} & 0 & {{1 \mathord{\left/ {\vphantom {1 2}} \right. \kern-\nulldelimiterspace} 2}} & 0 & 0 \\ 0 & {{1 \mathord{\left/ {\vphantom {1 3}} \right. \kern-\nulldelimiterspace} 3}} & 0 & {{1 \mathord{\left/ {\vphantom {1 3}} \right. \kern-\nulldelimiterspace} 3}} & {{1 \mathord{\left/ {\vphantom {1 3}} \right. \kern-\nulldelimiterspace} 3}} \\ 0 & 0 & {{1 \mathord{\left/ {\vphantom {1 2}} \right. \kern-\nulldelimiterspace} 2}} & 0 & {{1 \mathord{\left/ {\vphantom {1 2}} \right. \kern-\nulldelimiterspace} 2}} \\ 0 & 0 & {{1 \mathord{\left/ {\vphantom {1 2}} \right. \kern-\nulldelimiterspace} 2}} & {{1 \mathord{\left/ {\vphantom {1 2}} \right. \kern-\nulldelimiterspace} 2}} & 0 \\ \end{array} } \right] $$

The multiplication of 5 $\times$ 5 row standardized matrix, W ^s^, with 5 $\times$ 1 vector of y values taken by each region produces wy commonly called spatial lag vector of the dependent variable, as illustrated below:

$$ Wy = \left[ {\begin{array}{*{20}c} 0 & 1 & 0 & 0 & 0 \\ {{1 \mathord{\left/ {\vphantom {1 2}} \right. \kern-\nulldelimiterspace} 2}} & 0 & {{1 \mathord{\left/ {\vphantom {1 2}} \right. \kern-\nulldelimiterspace} 2}} & 0 & 0 \\ 0 & {{1 \mathord{\left/ {\vphantom {1 3}} \right. \kern-\nulldelimiterspace} 3}} & 0 & {{1 \mathord{\left/ {\vphantom {1 3}} \right. \kern-\nulldelimiterspace} 3}} & {{1 \mathord{\left/ {\vphantom {1 3}} \right. \kern-\nulldelimiterspace} 3}} \\ 0 & 0 & {{1 \mathord{\left/ {\vphantom {1 2}} \right. \kern-\nulldelimiterspace} 2}} & 0 & {{1 \mathord{\left/ {\vphantom {1 2}} \right. \kern-\nulldelimiterspace} 2}} \\ 0 & 0 & {{1 \mathord{\left/ {\vphantom {1 2}} \right. \kern-\nulldelimiterspace} 2}} & {{1 \mathord{\left/ {\vphantom {1 2}} \right. \kern-\nulldelimiterspace} 2}} & 0 \\ \end{array} } \right]\,\,\left[ {\begin{array}{*{20}c} {y_{1} } \\ {y_{2} } \\ {y_{3} } \\ {y_{4} } \\ {y_{5} } \\ \end{array} } \right] = \left[ {\begin{array}{*{20}c} {y_{2} } \\ {0.5y_{1} + 0.5y_{3} } \\ {0.3y_{2} + 0.3y_{4} + 0.3y_{5} } \\ {0.5y_{3} + 0.5y_{5} } \\ {0.5y_{3} + 0.5y_{4} } \\ \end{array} } \right] $$

## Model formulation

If the expression in Eq. (1) is restated in a matrix form and the error structure takes the form $u=\rho Wy+\varepsilon$ or $u=\lambda Wu+\varepsilon$, the two resulting models are called spatial lag and spatial error models. Mathematically, they are given as:

4 $$y=\rho Wy+X\beta +\varepsilon $$

5 $$y=X\beta +u$$

where $$u=\lambda Wu+\varepsilon $$ and $$\varepsilon \sim N(0, {\sigma }^{2}{I}_{n})$$

From (4) the implied DGP is given as:

6 $$y=({\mathrm{I}}_{\mathrm{n}}-\rho W{)}^{-1}X\beta +({\mathrm{I}}_{\mathrm{n}}-\rho W{)}^{-1}\varepsilon $$

The model statement in (6) can be “interpreted as indicating that the expected value of each observation ${y}_{i}$ will depend on the mean value $X\beta$ plus a linear combination of values taken by neighbouring observations scaled by the dependence parameter,$\rho$^28^. The infinite series expansion of $({\mathrm{I}}_{\mathrm{n}}-\rho W{)}^{-1}$ is given in (7) according to^28,29^.

7 $$({\mathrm{I}}_{\mathrm{n}}-\rho W{)}^{-1}={\mathrm{I}}_{\mathrm{n}}+\rho W+{{\rho }^{2}W}^{2}+{{\rho }^{3}W}^{3}\dots $$

Hence, the re-expression of SAR DGP for vector y shown in Eq. (6) follows thus:

8 $$y=X\beta +\rho WX\beta +{\rho }^{2}{W}^{2}X\beta +{\rho }^{3}{W}^{3}X\beta \dots +\varepsilon +\rho W\varepsilon +{\rho }^{2}{W}^{2}\varepsilon +{\rho }^{3}{W}^{3}\varepsilon +\dots $$

The ideal expressed in (8) is that rows of the weight matrix $W$ are constructed to signify first-order contiguous neighbours. Equally, matrix ${W}^{2}$ reflects second-order contiguous neighbours, that is, those that are neighbours to the first-order neighbours. This connotes neighbour of the neighbour to an observation $i$ includes observation itself. Hence, ${W}^{2}$ has positive elements on the diagonal. “The implication of this is that higher-order spatial lags can lead to a connectivity relation for an observation $i$ such that ${W}^{2}\varepsilon$ will extract observations from the vector $\varepsilon$ that point back to itself”. This is in stark contrast with the conventional independence relation in ordinary least-squares regression where the Gauss-Markov assumption rules out dependence observation of ${\varepsilon }_{i}$ on other observations $j$, by assuming zero covariances between $i$ and $j$ in the data generating process^30^.

The DGP for spatial error model shown in (5) where the disturbances exhibit spatial dependence is given as

9 $$y=X\beta +\varepsilon +\lambda W\varepsilon +{\lambda }^{2}{W}^{2}\varepsilon +{\lambda }^{3}{W}^{3}\varepsilon +\dots $$

From the foregoing, it is clear that in the spatial lag model, the spatially lagged dependent variable captures the spatial dependence between the cross-sectional units whereas in the spatial error model, the spatial autocorrelation term captures the spatial dependence^31^. Posited two economic arguments in support of SEM over SAR and Spatial Durbin models. Firstly, they argued that the SEM model constitutes a fuller representation of the spatial dependence than SAR and spatial Durbin model (an extension of the SAR model in which the lag effect of the dependent and independents variables are included in the model specification). This is because with the SEM model the spatial dependence can be influenced by other considerations in addition to shocks to the spatially lagged dependent variable. Secondly, they considered a situation where the total demand is disaggregated into two categories 1 and 2, a Wald test of the whole set of coefficients from the model for category 1 against 2, the set of coefficients from the model for category 2—which is necessary to establish if there is more to be learnt from disaggregating the data can be performed with easy on spatial error model. This is because the set of explanatory variables will be the same for a pair of SEM models. However, such a test cannot be performed on a pair of SAR models or a pair of spatial Durbin model because the spatially lagged dependent variables will differ in the two models. Though an exhaustive discussion of the spatial Durbin model will not be considered in this study, yet it must be remarked that this kind of model was developed with motivation to account for spatial dependence in the independent variable. This rationale stems from the idea that dependence in spatial relationships does not only occur in the dependent variable but also in the explanatory variables.

Another representative of the family of spatial regression models that is of interest in this study is the one that includes both endogenous interaction impacts and interaction effects among the error terms. Based on^31^ and related works, this model type was advocated for in the World Conference of the Spatial Econometrics Association held in 2017^32^. Labelled this model the Kelejian–Prucha model after their article in 1998 since they were the first to set out an estimation method for this model, also when the spatial weights matrix used to specify the spatial lag and the spatial error structure is the same. Whereas it was named Spatial Autoregressive with additional Autoregressive error structure (SARAR) or Cliff-Ord type spatial model by^31^ themselves^33^. Termed the model “Spatial Autoregressive Confused” (SAC)^17^. The specification takes the form:

10 $$y=\rho {W}_{1}y+X\beta +u$$

where $$u=\lambda {W}_{2}u+\varepsilon $$, $$\varepsilon \sim N(0, {\sigma }^{2}{I}_{n})$$

The DGP of the model is of the form:

11 $$y=({\mathrm{I}}_{\mathrm{n}}-\rho {W}_{1}{)}^{-1}X\beta +({\mathrm{I}}_{\mathrm{n}}-\rho {W}_{1}{)}^{-1}({\mathrm{I}}_{\mathrm{n}}-\lambda {W}_{2}{)}^{-1}\varepsilon $$

At first glance, the specification appears to represent a mixture of both spatial dependences in the dependent variable and the disturbances represented by W_1_y and W_2_u, respectively. A more formal examination of specification produce from a mixture of spatial dependence in the dependent variable and the disturbances is provided by^34^.

## Result and discussion

### Variables’ description and data screening for spatial autocorrelation

Most statistical procedures and inferences usually work well on the assumption of normality of the data. The data used for the study were explored for this essential criterion. The statistical properties of the variables are presented in Tables 2 and it showed that the spread of the variables from their central level is substantial and hence the high level of coefficient of variation. However, northing is less dispersed (CV = 32%) when compared with precipitation, easting and elevation. There is a moderate level of skewness in the variable except for elevation. Due to the instability and skewness tendencies, all the variables were log-transformed, and this enhanced their statistical properties. For instance, the skewness decreased for all the variables while the leptokurtic and platykurtic nature of the variables became smoothed to realize approximately normally distributed variables. The low-level interrelationship among the independent variables depicted by the correlation matrix is a signal that the selected variables passed the Gauss Markov assumption of absence of multicollinearity. Also worthy of note is the relationship of the selected independent variables with precipitation. Negative correlation was established between precipation and northing which indicated that precipitation decreased from south towards the north. However, positive correlation existed between easting and northing and this indicated that precipitation increase from west towards the east^35^.

**Table 2:** Statistical properties of the variables.

Statistics	Precipitation	Northing	Easting	Elevation
**Before transformation**				
Mean	100.78	479,291	809,128.8	267.5
Std. Dev	42.27	221,803.6	256,689	206.22
Minimum	35.89	171,369	317,099	4
Maximum	186.77	830,299	1,500,000	1344
Skewness	0.41	0.18	0.47	1.22
Kurtosis	2.02	1.51	2.91	6.3
**After transformation**				
Mean	4.52	13.55	12.96	5.13
Std. Dev	0.44	0.33	0.51	1.18
Minimum	3.58	12.67	12.05	1.39
Maximum	5.23	14.22	13.63	7.2
Skewness	− 0.2	− 0.34	− 0.2	− 1.11
Kurtosis	2.04	2.78	1.61	3.55

	LnPrecipitation	Lnnorthing	Lneasting	LnElevation
**Correlation matrix**				
LnPrecipitation	1			
LnNorthing	− 0.203**	1		
LnEasting	0.195**	0.233**	1	
LnElevation	− 0.665**	0.227**	− 0.127*	1

**. Correlation is significant at the 0.01 level (2-tailed)

*. Correlation is significant at the 0.05 level (2-tailed)

**Figure 2:**
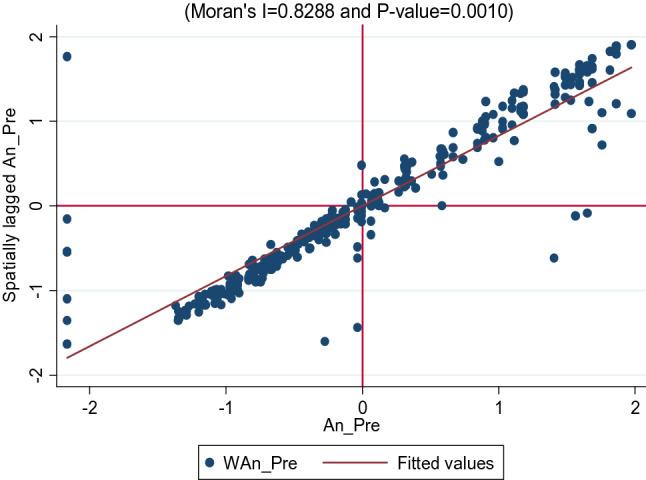
Moran’s I scatter plot for annual precipitation.

The dependent variable was first diagnosed for spatial autocorrelation using Moran’s I scatter plot (Fig. 2)^36^. The data in the plot are standardized so that units on the graph are conveyed in standard deviations from the mean^37^. The horizontal axis demonstrates the standardized value of precipitation for a county, the vertical axis shows the standardized value of the average precipitation (WAn_P_re), for that county’s neighbours as defined by the order one queen weights matrix. The slope of the regression line through these points expresses the global Moran’s I and this is estimated to be 0.8288 with a *p* value of 0.0010 in this study^37,38^.

Similarly, a global measure of spatial autocorrelation was computed for the variables using both Moran’s and Greary’s C. As shown in Table 3, all the variables have positive and significant spatial autocorrelation, and this implies that similar values of each variable occur near their contiguous locations. The upper right quadrant of the Moran’s I scatter plot showed those counties with above-average precipitation and share above-average precipitation with neighbouring counties (high-high)^39,40^. These are regarded as the hot spot locations while the lower left quadrant which shows counties with below-average precipitation values and neighbours also with below-average values (low-low) is the cold spot locations^39,40^. The lower right quadrant displays counties with above-average precipitation surrounded by counties with below-average values (high-low), and the upper left quadrant contains the reverse (low–high)^41,42^. They are called spatial outliers.

**Table 3:** Measures of global spatial autocorrelation.

	I	Z	*p* value	C	Z	*p* value
Variables		Moran’s I			Geary’s c	
Precipitation	0.83	25.52	0.000	0.17	− 24.50	0.000
Northing	0.92	28.47	0.000	0.08	− 26.97	0.000
Easting	0.64	19.83	0.000	0.36	− 19.18	0.000
Elevation	0.13	5.28	0.099	0.82	− 1.83	0.067

**Figure 3:**
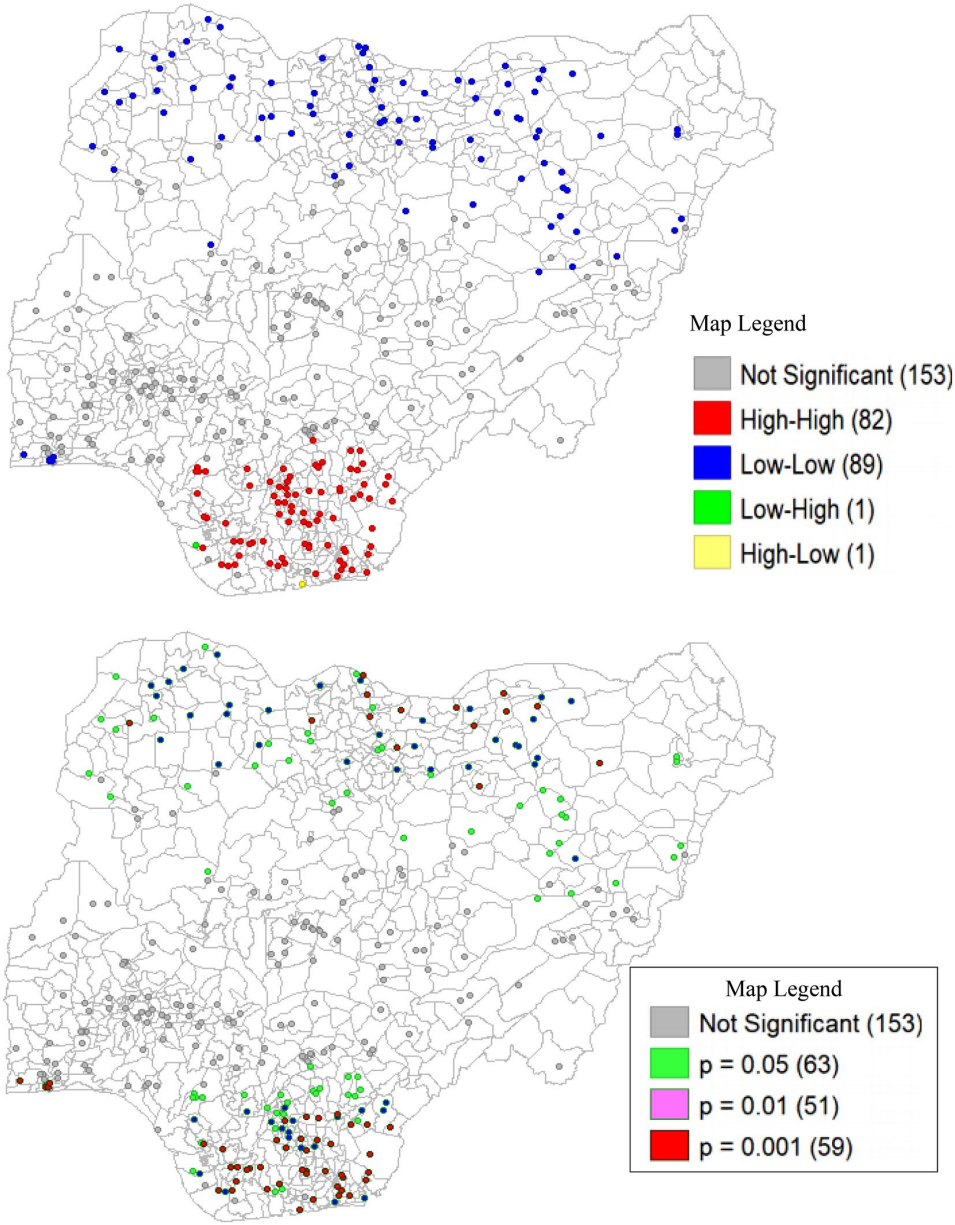
LISA (top) and significant (bottom) maps showing the spatial distribution of precipitation.

Figure 3 (top, bottom) is a Local Indicator of Spatial Autocorrelation (LISA) and significant maps, respectively. These maps shed light on the clustering suspected in the Moran’s I scatter plot. The red colour in this figure (top) depicted the hot spot location of precipitation and there 82 of such locations in our data predominantly in the Southern regions of the country. In the same vein, the blue colour represented the cold spot i.e., clusters with low levels of precipitation and there are 89 points with such attribute in the dataset. Additional information provided by the significant map (Fig. 3, bottom) indicated that 63 (19.3%), 51 (15.6%) and 59 (18.10%) of the sample observation showed statistically significant local spatial autocorrelation at 5%, 1% and 0.1% level of significance, respectively.

### Spatial variability and continuity

**Figure 4:**
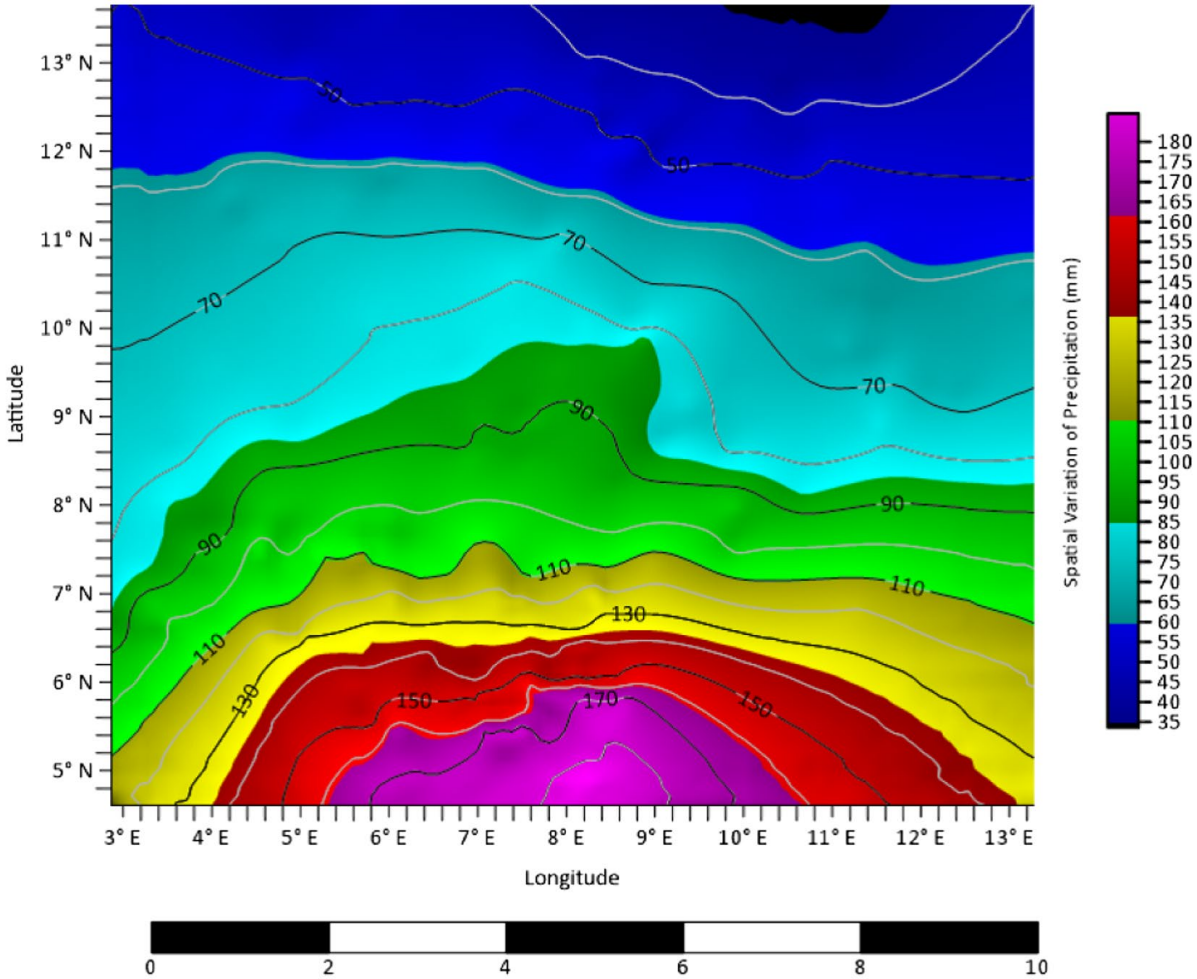
Precipitation surface map (mm).

The knowledge of the spatial clustering in the data from the previous subsection necessitated further exploration with geostatistical tools. The 3-D surface map presented in Fig. 4 unambiguously described the interrelationship of precipitation in the study area with other geographic variables. it was noted from the map that precipitation values increase with decreases in the height above the sea level (elevation) and latitudinal values whereas longitudinal values have an irregular pattern as one move from west to east. This result implies that locations with high latitudes tend to experience low precipitation while those in the low latitudes have high precipitation. Equally, the high altitudes locations have high precipitation as against those in the low altitudes. From geography perspectives, Latitudes is simply a measure of how far one is from the equator while elevation measure how high one is above the sea level, so the locations that are far from the equator or have high elevation are prone to cold weather or climate compare with those in the low latitudes. This explains the uneven distribution of precipitation in the northern and southern part of Nigeria. The locations in the core north of the country are at high latitudes and altitudes and by implication far from the equator. This result is of great relevance in agriculture and water resource management.

**Figure 5:**
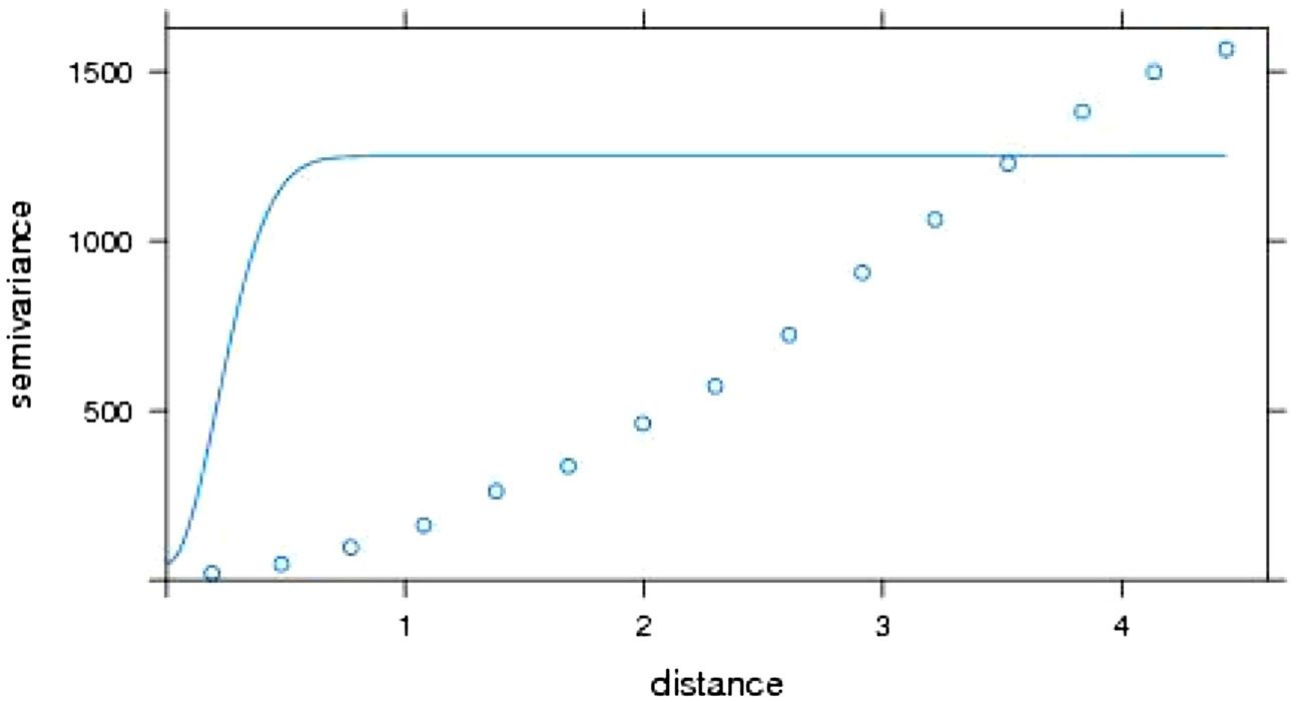
Variogram plot for precipitation^29^.

Further Gaussian model experimental variogram was fitted to the precipitation data and the basic spatial parameters were calculated using R statistical software (Fig. 5). The calculated parameters were a nugget, range and sill. The nugget is the value at which the model intercept y-axis and it can be interpreted as the variance at zero distance between a unit and its neighbour, the range is the distance where the model first flattens, and this can be interpreted as the distance where the value of one variable becomes spatially independent^43^ while the value at which the model attains the range is called the sill and it is interpreted at the lag distance between the measurements at which one value for a variable does not influence neighbouring values (discontinuity). The nugget, range, and the sill for the variable under investigation were found to be 50, 0.3 and 1200, respectively. The ratio of the nugget variance to the sill is the spatial coefficient parameter^43,44^ and for this study, it was estimated to be 4.2%. Following the classification of^10,45^, this value indicated strong spatial autocorrelation in the precipitation series.

A diagnostic check was conducted for spatial dependence in OLS regression after it has been confirmed that spatial clustering is present in the dataset. The aim here is to unearth the type of spatial effect present in the dataset and model as appropriate. To achieve this, the Lagrange Multiplier specification test for spatial lag and error (LM_LAG_ and LM_ERROR_ ) was conducted on the residuals extracted from the fitted OLS regression^42,46^. If neither the LM_LAG_ nor LM_ERROR_ statistics rejects the null hypothesis then OLS is appropriate. If one of the LM statistics rejects the null hypothesis, but the other does not then the decision is straightforward. The alternative spatial regression model that matches the test statistic that rejects the null hypothesis^47,48^. When there is conflict, that is when both LM_LAG_ and LM_ERROR_ statistics rejects the null hypothesis, to select an adequate model, focus shift to robust forms of the test statistics^40–42,42,48^. Typically, only one of them will be significant (for example LM_ERROR_ as in Table 4), or one will be more significant than the other. It is important to note that when both robust forms are significant, a model matching the (most) significant statistics is estimated. When both are highly significant, the model with the larger value of test statistics is considered appropriate however there may be other causes of misspecification^40^.

**Table 4:** Diagnostic tests for spatial dependence in OLS regression.

Test	Statistic	df	*p* value
	Spatial error		
Moran's I	17.576	1	0.000
Lagrange multiplier	287.857	1	0.000
Robust Lagrange Multiplier	30.101		0.000
	SPATIAL LAG		
Lagrange multiplier	258.502	1	0.000
Robust Lagrange multiplier	0.746	1	0.388
	SARMA		
Lagrange multiplier	288.603	1	0.000

The LM-SARMA will tend to be significant when neither of them is appropriate^23^. From the foregoing illustration, the SEM model was appropriate if a choice was to be made between it and the SAR counterpart. Observe that both the LM_LAG_ and LM_ERROR_ were significant but the robust form of the spatial lag model was insignificant^40^.

To enhance further comparison, five models were estimated of which the first is non-spatial while the rest four take spatial specification form. The models included traditional linear regression, spatial lag (SAR), spatial error (SEM), spatial Durbin and SARAR. The spatial models were estimated by maximizing the corresponding likelihood while the non-spatial model was estimated by the OLS method.

Table 5 reported the summary of the result from the five models. Columns 1, 2, 3, 4 and 5 indicated the estimates obtained from OLS, SAR, SEM, Durbin and SARAR models, respectively. A close look at the result showed that the OLS, SAR and SEM models estimates were alike in term of the sign and significance but differs in term of the sign when compared with Durbin and SARAR estimates. However, the OLS was characterized with either under or overestimation of the coefficient. For instance, OLS over-estimated the coefficient for easting by 42.9% compared with the SAR model while it is over-estimating this coefficient by 8.6%, 13.6% and 9.5%, respectively when compared with ”SEM”, DURBIN and ”SARAR” models. This inflation or deflation of the coefficient was not surprising because of the significance of the spatial dependence in the dataset. This result also buttresses the position of^49–51^ that nonspatial OLS is devastating and to be avoided unless interdependence is known to be very weak or nonexistent.

**Table 5:** Predictors of annual precipitation and selection criteria estimates.

Independent variables	OLS	SAR	SEM	DURBIN	SARAR
LogEasting	0.195*	0.137*	0.214*	0.226*	0.216*
LogNorthing	0.050	0.043	0.017	0.006	− 0.010
LogElevation	− 0.050	− 0.038	− 0.004	0.000	0.008
wx_LogEasting				− 0.206	
wx_LogNorthing				0.030	
wx_LogElevation				− 0.016	
Intercept	1.467***	− 0.940***	1.443***	0.325***	3.942***
**Rho (**${\varvec{\rho}})$		0.726***		0.777***	− 0.496***
**Lambda (**$\lambda )$			0.779***		0.919***
**Model selection criteria**					
Sigma		0.569	0.547	0.547	0.501
AIC	787.523	609.734	591.883	597.636	581.789
BIC	802.671	632.456	614.605	631.718	608.297
**Weight Matrix (Queen Order 1)**	${\varvec{N}}{\varvec{o}}{\varvec{n}}{\varvec{e}}$	$326\times 326$	$326\times 326$	$326\times 326$	$326\times 326$

* *p* < 0.10, ** *p* < 0.05, *** *p* < 0.01.

The spatial effect parameters ($\lambda$ and $\rho$) in the spatial models are found to be highly significant. The SAR, SEM, and DURBIN have the spatial effect of $\rho$= 0:726, *p* < 0.01, λ = 0:779, *p* < 0.01 and $\rho$= 0:777, *p* < 0.01, respectively. The SAR coefficient indicates that the association between the dependent variable and its contiguous counties, the SEM coefficient, show the association of the error term with the neighbouring observation while the DURBIN coefficient gives the idea on the level of dependence of the spatial lag of the independent variables. In the case of SARAR, $\rho$ is negative and significant while λ is positive and significant ( $\rho$= 0:496, *p* < 0.01;λ = 0:919, *p* < 0.01). The spatial effect coefficients of the SARAR model gives a level of spatial association in the dependent variable and its neighbours as well as the error and its connected regions.

The model selection criteria statistics are presented in the last panel of Table 5. Using the two criteria, the OLS value is highest among other models. This indicates that OLS performs poorly in the presence of spatial clustering and that the spatial model will produce a robust estimate of the parameter. This finding is in agreement with the earlier report by^3^ that spatial models are superior to nonspatial OLS when a spatial effect is detected in the model.

Overall, the SARAR model produced a better fit for the regression relation because its model selection criteria values were smallest compared with other spatial models. Based on the selected model, only the easting significantly explained precipitation. However, it was noted that easting and elevation exerted a positive impact on precipitation while northing impact was negative. It implies that precipitation increase with a corresponding increase in easting and elevation whereas it depreciates as northing appreciate. The positive effect of easting on precipitation indicated there would an increase in precipitation value for any unit movement from west towards the east, while the negative effect of northing (though not significant) depicted a decrease in precipitation for any unit movement from south towards the north.

## Conclusion

This study discussed the rationale for an alternate technique to the conventional regression proposition of independence of observation and applied the approach to building a regression relationship between three predictors (Easting, Northing and Elevation) and precipitation. Exploratory data analysis tools were used to detect spatial autocorrelation, hot spot and a cold spot of precipitation in the study area. The results agreed with previous studies on the superiority of spatial models over OLS. On the premise that spatial models achieved significant improvement over their traditional counterpart, it indicated that spatial models were not only the correct specification but also a more effective approach. However, this study added that a spatial model that simultaneously accounted for spatial effect in the dependent variable and error term provides a better fit compared with the SAR and SEM used in the earlier studies of^4,52^ for precipitation modelling.

The spatial modelling approach discussed is quite rich and provided the basis for choosing a particular regression specification, unlike the orthodox framework where the model is imposed on the data without investigating what the data reveal about itself and how it should be modelled. Data exploration is very important, and it is one of the key ways of avoiding misspecification and misleading result.

## Materials and methods

### Study area

**Figure 6:**
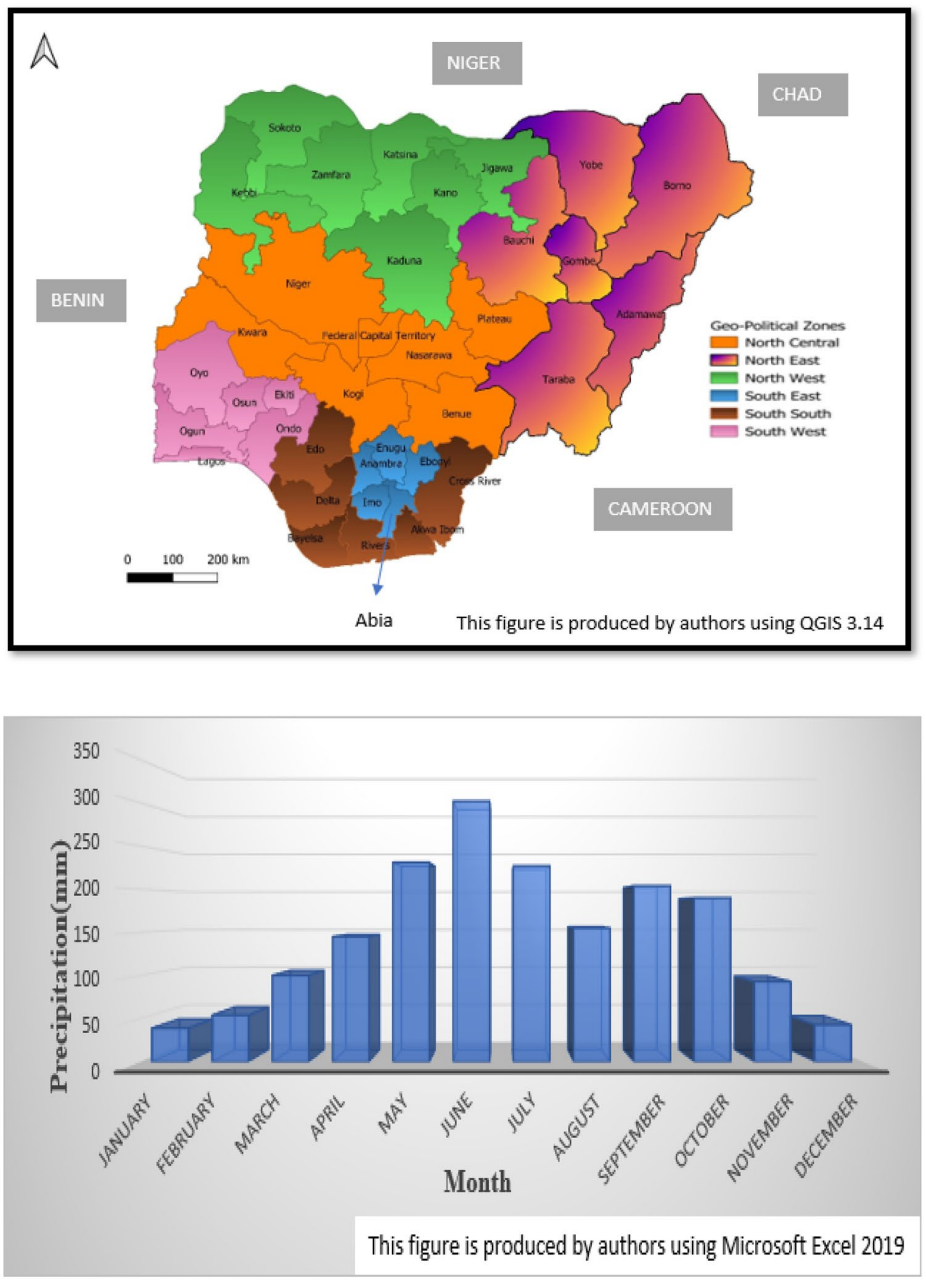
Map of Nigeria Showing the Six Geopolitical Zones (top) and Monthly distribution of Precipitation of Nigeria (bottom).

The study was conducted in Nigeria, a country in the Sub-Saharan region. The country is situated in West Africa and bordered in the North and Northeast by the Niger Republic and the Republic of Chad, respectively. Also, it shared a boundary with the Republic of Cameroon and the Republic of Benin in the East and West, respectively. To the South, Nigeria is bordered by approximately 850 kms of the Atlantic Ocean, stretching from Badagry in the West to Rio del Rey in the East. It lies within latitudes of (4 14 N) and longitudes of (3 13 E) with a total land area of 923,768 square kilometres (Fig. 6, top)^53^. Nigeria has two distinct seasons: dry and wet. These seasons are based on the proximity of each region or location in the nation to the Intertropical Convergence Zone (ITCZ). The dry season is between October to March while the wet season is between April to September annually with June and July often the wettest (Fig. 6, bottom) (online resources: https://www.britannica.com/place/Nigeria/Climate).

### Data and methods

The data was sourced from the Nigeria Malaria Indicator Survey (MIS) of 2015. The suitability of MIS for the study was based on its national representativeness and provision of geo-referenced information required for spatial modelling. The geographical covariates were provided in a shapefile format and consist of climatic variables for 329 clusters. The precipitation data for the year 2015 for all the 329 clusters and their respective coordinates were extracted but only 326 observations were suitable for analysis after removing inconsistent cases^3,4,35,52^. Modelled precipitation as a function of easting, northing and elevation and this specification is adopted in this study due to limited data. The easting and northing variables for each cluster were obtained by transforming the latitudes and longitudes of these 326 locations to Standard Universal Transverse Mercator (UTM) using ”PAleontological STatistics” (PAST) software. By definition, the **northing value** is the distance of the position from the **equator** in meters while the **easting value** is the distance from the **central meridian** (longitude) of the used UTM zone (the study area has three UTM zones, namely, 31, 32 and 33). Before model estimation, the variables were diagnosed for spatial variability and clustering. Various exploratory tools were used to describe and visualize spatial distributions; identify uneven locations or spatial outliers; discover pattern of association, cold or hot spots^39,41–43,54,55^. Firstly, a 3-D surface contour map was used to examine the spatial variability of precipitation along the lines of longitude and latitude as well as its behaviour relative to a height above sea level. Secondly, the Variogram plot was used to study the precipitation data for a possible tendency of spatial dependence and discontinuity.

The spatial weighting matrix was created by employing GeoDa software using the queen definition of neighbour discussed in Section "Spatial data concept and model formulation" and formatted as ”spmat” object and imported to STATA software for further exploration of the data. Basic information about the spatial weighting matrix is presented in Table 6. The number of neighbours among the clusters range between 2 and 12 links with each county having 6 neighbours on average and a total of 1920. The 3-D surface contour map, Variogram plot, weighting matrix creation, and regression modelling were carried out using Surfer, R, GeoDA and STATA statistical software packages, respectively. Each software was chosen based on the ease of undertaken assigned task and the time of execution.

**Table 6:** Summary of spatial-weighting object, W.

Matrix	Description
Dimension	326 × 326
Total	1920
Minimum	2
Mean	5.889751
Maximum	12

The weighting matrix created by ”spmat” command was used to produce a cluster map for precipitation and thereafter was converted to a text file by using the “export” command in STATA. The resulting text file was saved as “dta” file and imported as a row standardized weighting matrix of ”spatwmat” object. This ”spatwmat” weighting matrix format was used for the global and local indicator of spatial autocorrelation as well as to produce Moran’s I scatter plot.

12 $$ \left. {\begin{array}{*{20}l} {y = pWy + X\beta + WX\theta + u} \hfill \\ {u = \lambda Wu + \varepsilon } \hfill \\ {\left| \lambda \right| < 1, \left| \rho \right| < 1,\left| \theta \right| < 1} \hfill \\ \end{array} } \right\} $$

The general spatial regression expressed in Eq. (12) was transformed into five different models by imposing zero conditions on the parameters Rho $(\rho )$, Lamda $(\lambda )$ and Theta $(\theta )$. This produces four spatial and non-spatial regression models. When each $\rho$, $\lambda$ and $\theta$ are zeros, the traditional OLS model (Eq. 1) is recovered. Also, SAR and SEM expressed in Eqs. 3 and 4 surfaced when λ = 0 , $\theta$= 0 and $\rho$= 0 , $\theta$= 0, respectively. DURBIN and SARAR models resulted in the condition that λ = 0 and $\theta$= 0, respectively. In this expression, y = precipitation is the dependent variable, X is a vector of exogenous variables which are northing, easting and elevation. λ,$\rho$ and $\theta$ are the coefficients for spatial lagged dependent, the error term and the independent variables while u is the independent and identically distributed error term. All the spatial models were estimated using the”spmlreg” STATA module which is based on the maximum likelihood method.
